# Prediction of Loss of Muscle Mass in Sarcopenia Using Ultrasonic Diaphragm Excursion

**DOI:** 10.1155/2021/4754705

**Published:** 2021-12-06

**Authors:** Bin Zeng, Shaochong He, Hao Lu, Guiyin Liang, Xiaosong Ben, Wenzhao Zhong, Mingsheng Zhang, Hao Wang

**Affiliations:** ^1^Department of Physical Medicine and Rehabilitation, Guangdong Provincial People's Hospital, Guangdong Academy of Medical Sciences, Guangzhou, China; ^2^Department of Anesthesiology, The First Affiliated Hospital, Jinan University, Guangzhou 510630, China; ^3^Department of Thoracic Surgery, Guangdong Provincial People's Hospital, Guangdong Academy of Medical Sciences, Guangzhou, China; ^4^Guangdong Lung Cancer Institute, Guangdong Provincial People's Hospital, Guangdong Academy of Medical Sciences, Guangzhou, China

## Abstract

**Background:**

The diagnosis of sarcopenia is based on the mass and function of appendicular skeletal muscle. It is not clear whether diaphragm excursion is related to muscle mass loss. We try to fill the gap by measuring ultrasonic diaphragm excursion during quiet breathing (Dq) and forced deep breathing (Df) and test whether they could predict the muscle mass loss in sarcopenia.

**Methods:**

The subjects are recruited from the elderly patients diagnosed with pulmonary nodules in community physical examination. According to the definition, the subjects were divided into group A (who did not meet the diagnostic criteria for muscle mass loss in sarcopenia) and group B (who met the criteria). Participants were assessed for ultrasonic diaphragm excursion, pulmonary function, and cardiopulmonary exercise testing. Logistic regression was used to assess the correlation between right diaphragm excursion and skeletal muscle mass, and receiver-operating characteristic curve (ROC) was applied to determine the best threshold.

**Results:**

We recruited 64 elderly participants: 52 in group A (39 males) and 12 in group B (8 males). The Df in group A were higher than in group B (6.02 (5.44–6.60) vs. 4.31 (3.53–5.09) cm, *P*=0.008). The difference also exists in FVC, FEV1.0, PEF, Pimax, WRmax, and VO_2_max, but neither in Dq. Logical regression showed that Df was negatively related to muscle mass (*B* = −0.525, OR = 0.591 (0.378–0.926), *P*=0.022), even after adjusted age. Based on ROC, a cutoff value of 5.27 cm (AUC = 0.7783, *P*=0.0028) was selected, and Df ≤ 5.27 cm indicates the increase in odds of existing muscle mass loss.

**Conclusion:**

Ultrasonic diaphragm excursion in forced deep breath is helpful for predicting muscle mass loss in sarcopenia. The trial is registered with ChiCTR1800019742.

## 1. Introduction

Sarcopenia is a syndrome characterized by the skeletal muscle mass loss and dysfunction. The disease affects not only peripheral muscle groups but also respiratory muscle groups. As the primary inspiratory muscle, the diaphragm responds 75% of the inspiratory volume, which has a significant impact on the exercise ability. Previous studies have confirmed the mechanism of diaphragm mass loss, which were accompanied with aging and chronic diseases, was different from peripheral muscles [1]. The diagnostic criteria of sarcopenia were primarily based on the decrease of appendicular muscle mass and function. However, the change of diaphragm mass and function by aging is uncertain.

Ultrasound has been applied in evaluating the mass and function of peripheral muscles and has been confirmed by several valuable clinical indicators. As an important parameter of diaphragm, its excursion is regarded to be related with individual lung function, respiratory muscle strength, and weaning outcome of patients undergoing long-term mechanical ventilation.

So, we perform the present study in community elderly to prove that ultrasonic diaphragm excursion would decline accompanied with muscle mass loss and make clear its clinical value in predicting the muscle mass loss in sarcopenia.

## 2. Methods

### 2.1. Study Design

This study is part of a randomized controlled clinical trial conducted by Guangdong Provincial People's Hospital (Guangdong Academy of Medical Sciences). It has been approved by the Medical Research Ethics Committee of Guangdong Provincial People's Hospital (Guangdong Academy of Medical Sciences) and registered in China Clinical Trial Registry (ChiCTR1800019742). This study includes elderly (≥60 years), diagnosed pulmonary nodules or lung cancer by routine physical examination in community, without respiratory or tumor consumption symptoms. We exclude the patients with extrapulmonary metastasis before or during pneumonectomy. All subjects have submitted written informed consent at the beginning of the study. The sex, age, height, weight, smoking status, medical history, and medication history of the participants are collected.

### 2.2. Pulmonary Function Testing

The pulmonary function testing was performed in our hospital (HU-101, CHEST, Japan). The absolute values and their percentage to the predicted values, including preoperative vital capacity (VC), forced vital capacity (FVC), forced expiratory volume in one second (FEV1.0), FEV1.0/FVC, maximum midexpiratory flow (MMEF), and peak expiratory flow (PEF), were recorded.

### 2.3. Respiratory Muscle Strength Testing

The index of respiratory muscle strength were measured by a hand-held breathing pressure measuring instrument (X1, XEEK, China). According to the ATS statements, the patient sat and rested 3 minutes, slowly exhaled to the residual volume, and then quickly inhaled to the total lung volume, recorded the maximum inspiratory pressure (Pimax), or slowly inhaled to the total lung volume and quickly exhaled to the residual volume, recorded the maximum expiratory pressure (Pemax). Every index was measured three times, and the maximum one was recorded.

### 2.4. Ultrasonic Diaphragm Excursion Testing

**Figure 1:**
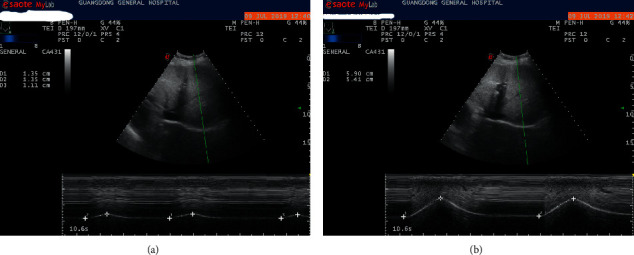
Diaphragm excursion in quiet (a) and forced deep breath (b).

The diaphragm excursion is measured by M-model ultrasonography (MyLab 70, XVision, Italy) and performed of the right hemidiaphragm in order to obtain clearer image. The probe was placed below the right subcostal position, angled 30° with the abdomen. The operator moved the probe back and forth between midclavicular and anterior axillary lines, so that the ultrasound beam was perpendicular to the posterior right hemidiaphragm. The diaphragm excursion (3.5 MHz, M-mode) in quiet breath and forced deep inspiration (from the residual volume to the total lung volume) was recorded as Dq and Df, respectively (Figure 1). Every parament was measured five times, and the maximum value was recorded.

### 2.5. Human Body Composition Testing

Human body composition was measured by the bioelectrical impedance technique (MC-180, Belia Body Component Analysis Instrument, Japan). After cleaned the palms and feet with alcohol, the subjects stood on the metal pedal of the analyzer and held metal handle for 30 seconds. Body composition parameters including body mass index, muscle mass, fat mass, muscle mass of limbs, and visceral fat mass were recorded.

### 2.6. Cardiopulmonary Exercise Testing

Cardiopulmonary exercise testing (CPET) was evaluated by symptom-limited maximal incremental exercise testing protocol (Metalyzer, Custo-Med, Germany). According to ATS statements, the incremental load per minute was adjusted according to the predicted maximum power of the subjects. The maximal power (WRmax), maximal oxygen uptake (VO_2_max), maximal kilogram oxygen uptake (VO_2_max@Kg), maximal heart rate (HRmax), maximal minute ventilation (V_E_max), maximal tidal volume (V_T_max), and their percentage to the predicted value were recorded.

### 2.7. Definition of Sarcopenia

In order to define the muscle mass loss in sarcopenia, we recruited healthy volunteers from our interns (18–25 yeas) to measure their body composition parameters. Based on their measured outcome, we defined 2 standard deviations of the mean ASM/height^2^ of male or female interns as the cutoff points for elders' muscle mass loss, respectively.

### 2.8. Statistical Analysis Method

IBM SPSS 18 software was used for statistical analysis. The unpaired *t*-test was used to compare the difference between continuous variables. The Spearman test or logistic regression was used to analyze the correlation between the two variables. ROC curve was used to determine the optimal threshold value for predicting the muscle mass loss in the sarcopenia. All statistics assume that the significance level *α* is 0.05 and *β* is 0.1.

## 3. Results

**Figure 2:**
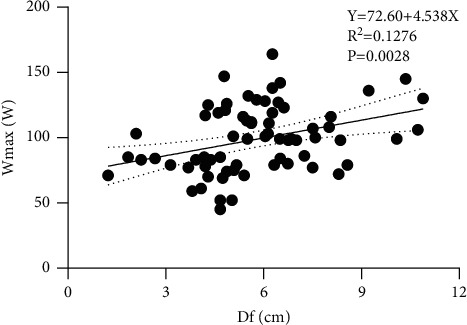
Linear regression analysis between Df and WRmax.

**Figure 3:**
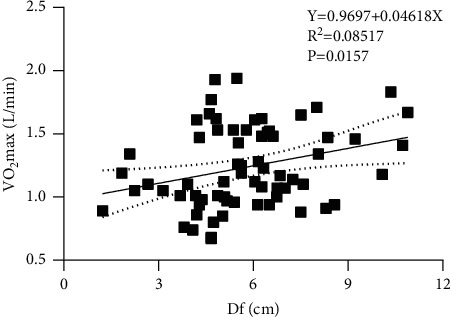
Linear regression analysis between Df and VO_2_max.

**Figure 4:**
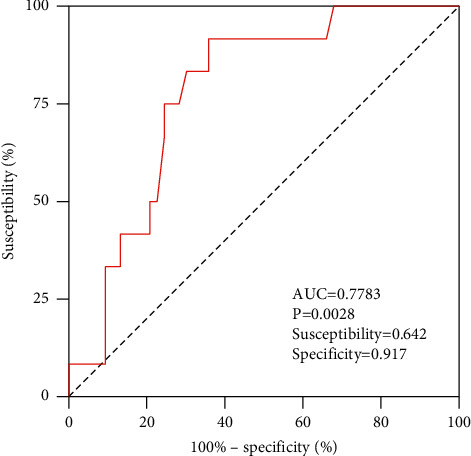
ROC for the predictive value of Df for muscle mass loss in sarcopenia.

1. A total of 44 young volunteers and 74 elderly volunteers were recruited, of which 68 elderly completed the body composition test, and 64 completed all tests including ultrasonic diaphragm excursion testing. Among young volunteers, the ASM/height^2^ for men was 8.01 ± 0.83 kg/m^2^ and for women was 6.61 ± 0.61 kg/m^2^. According to definition for muscle mass loss in sarcopenia, the cutoff points of ASM/height^2^ are ≤6.35 Kg/m^2^ for male and ≤5.39 Kg/m^2^ for female (Table 1).
2. There were 52 elders (39 males and 13 females) without muscle mass loss (group A) and 12 elders (8 males and 4 females) with muscle mass loss (group B).  Table 2 provides that Df in group A was higher than in group B (6.18 (5.57–6.79) vs. 4.36 (3.49–5.22) cm, *P*=0.008), but not for Dq. The pulmonary ventilation function and exercise performance in group A are better than group B. Furthermore, regression analysis indicates that Df can partly explain the variation of maximum power and maximum oxygen uptake in CPET (*R*^2^ = 0.1276, *P*=0.0028 and *R*^2^ = 0.0852, *P*=0.0157, respectively; Figures 2 and 3).
3. Logistics regression analysis was used to assess the correlation between sex, age, diaphragm excursion, and muscle mass loss.  Table 3 provides that age (model 1) and Df (model 2) were correlated with the muscle mass loss (*P*=0.040 and 0.013, respectively), while gender and Dq were not. After controlling for age, the Df (model 3) was still negatively correlated with muscle mass loss (*B* = −0.525, OR = 0.591 (0.378–0.926), *P*=0.022).
4. ROC was used to determine the optimal predictive value of Df for muscle mass loss in sarcopenia.  Figure 4 shows that the optimum threshold value of Df is 5.27 cm (AUC = 0.7783 (0.6537–0.9029), *P*=0.0028, Youden = 0.559, OR = 2.557).

## 4. Discussion

The findings of this study include the following. The decline of muscle mass in the elderly is accompanied by the decrease of diaphragm excursion in forced deep breath. Diaphragm excursion in forced deep breath is an independent predictor of maximal power and maximal oxygen consumption in the elderly. The decrease of diaphragm excursion in forced deep breath is helpful to predict muscle mass loss in the elderly, and the optimum predictive threshold is 5.27 cm.

Sarcopenia is a syndrome associated with aging. Because many factors such as sex, race, living habits, and other factors can directly affect its natural progress, most clinical guidelines or epidemiological studies of sarcopenia use the average value of healthy population in the same region and the race and gender group (20–30 years old) as the diagnostic threshold value [2, 3]. Therefore, this study included medical students in our department as a standard population to determine the diagnostic threshold of appendicular skeletal muscle mass index.

As a clinical accessibility parament, the repeatability and consistency of ultrasonic diaphragm excursion has been confirmed and has been widely used in the evaluation of diaphragm weakness or diaphragm dysfunction [4, 5]. Palkar et al. found that the diaphragm excursion in spontaneous respiratory was lower in patients with failed exudation than success, and the product of diaphragm excursion and inspiration time during spontaneous breathing was a better indicator for predicting the success rate of extubation [6]. Some authors also believe that the diaphragm excursion is a better predictive factor for successful extubation than the percentage of diaphragm thickening during inhalation [7]. In addition, the diaphragm excursion can reflect the effect of inspiratory muscle resistance training and is consistent with the changes of maximum inspiratory pressure, maximum expiratory pressure, and diaphragm thickening rate [8]. These studies all indicate that diaphragm excursion is an ideal index of diaphragm function. Our data show that elderly with muscle mass loss exhibit lower maximum diaphragm excursion, accompanied by decreased FVC, FEV1.0, PEF, Pimax, WRmax, VO_2_max, and other indicators, while diaphragm excursion in forced deep breath can also explain some of the WRmax and VO_2_max in CPET. This is consistent with previous research results.

Previous animal studies have confirmed that aging can not only affect the maximum diaphragmatic pressure, maximum muscle strength, and maximum endurance [9] but also change the composition of diaphragm muscle fiber [10]. According to the application of ultrasonography in the peripheral muscle group, Ticinesi et al. indicated that this technique can also be used to measure diaphragm excursion [11]. Scarlata et al. and colleagues have tested 100 healthy volunteers aged 40 ± 15 years and confirmed that diaphragm excursion in forced deep breath was negatively correlated with age (Spearman's coefficient = 0.272, *P*=0.006) [12]. Another small sample study involving patients with Duchenne muscular dystrophy also found a negative correlation between diaphragm excursion in forced deep breath and age (*R*^2^ = 0.68, *P* < 0.0001) [13]. So, we presume that aging may be negatively correlated with diaphragm excursion in forced deep breath, and our tests confirm this point. The decrease of diaphragm excursion in forced deep breath is correlated with muscle mass loss in the elderly, and the correlation is still statistically significant after controlling for age.

As the first study to explore the diagnostic value of diaphragm excursion for sarcopenia, this study has some limitations. First, the elderly in this study were all from patients with pulmonary nodules before pneumonectomy. Although we recruited elderly from communication and have excluded the patients with extrapulmonary metastasis to avoid the additional effect from lung cancer, there may be some differences from elderly well-being in communication. For example, the ratio of smoking and concomitant disease in the elderly we recruited may be more common. Those factors could affect diaphragm excursion. Second, as this study is a part of the clinical randomized controlled study, the sample size is small, so we have to limit our research to diaphragm excursion and cannot consider other factors including Pimax, Pemax, and PEF.

Nevertheless, the study results suggest that ultrasonic diaphragm excursion in forced deep breath is helpful in predicting muscle mass loss in the elderly. Larger cohort studies will be needed to confirm its diagnostic value for sarcopenia in the future.

## 5. Conclusion

Ultrasonic diaphragm excursion in forced deep breath is helpful for predicting muscle mass loss in sarcopenia.

## Figures and Tables

**Table 1:** Comparison of anthropometric parameters between different sex and age groups.

Variable	Male			Female		
	Young	Elderly	*P* value	Young	Elderly	*P* value
*n*	16	50		28	18	
Age (Y)	22.00 (20.95–23.05)	68.88 (67.37–70.39)	0.000	22.04 (21.63–22.44)	71.33 (68.99–73.67)	0.000
Height (m)	1.71 (1.68–1.73)	1.65 (1.64–1.67)	0.000	1.59 (1.57–1.61)	1.55 (1.52–1.58)	0.018
Weight (kg)	59.53 (54.82–62.24)	62.80 (59.56–66.05)	0.402	50.16 (47.43–52.88)	55.10 (51.62–58.58)	0.033
Muscle mass (kg)	48.20 (45.37–51.04)	47.21 (45.40–49.01)	0.526	34.83 (33.90–35.77)	34.41 (32.50–36.32)	0.645
ASM (kg)	23.36 (21.95–24.77)	20.55 (19.54–21.55)	0.005	16.64 (16.13–17.15)	13.39 (13.36–14.63)	0.000
ASM/height^2^ (kg/m^2^)	8.01 (7.57–8.45)	7.48 (7.18–7.78)	0.070	6.61 (6.38–6.85)	5.85 (5.61–6.10)	0.000

ASM, appendicular skeletal muscle mass.

**Table 2:** Comparison of body composition and diaphragm excursion between groups A and B.

Variable	Group A	Group B	*P* value
Dq (cm)	2.01 (1.71–2.29)	1.51 (0.92–2.10)	0.166
Df (cm)	6.18 (5.57–6.79)	4.36 (3.49–5.22)	0.008
FVC	2.83 (2.63–3.03)	2.31 (1.94–2.69)	0.011
FEV1.0	2.04 (1.87–2.21)	1.60 (1.32–1.86)	0.018
PEF	5.40 (4.81–5.99)	3.78 (3.03–4.54)	0.019
Pimax	64.20 (57.23–71.16)	45.45 (36.56–54.35)	0.017
WRmax	104.26 (97.08–111.44)	70.73 (61.71–79.75)	0.000
VO_2_max	1.30 (1.21–1.39)	0.88 (0.79–0.97)	0.000
V_E_max	53.12 (49.01–57.24)	41.05 (36.01–46.08)	0.000
V_T_max	2.61 (2.36–2.86)	2.03 (1.69–2.36)	0.001

Dq, ultrasonic diaphragm excursion during quiet breathing; Df, ultrasonic diaphragm excursion during forced deep breathing; FVC, forced vital capacity; FEV1.0, forced expiratory volume in one second; PEF, peak expiratory flow; Pimax, maximal inspiratory pressure; WRmax, maximal work rate; VO_2_max, maximal oxygen consumption; V_E_max, peak minute ventilation during exercise; V_T_max, peak tidal volume during exercise.

**Table 3:** Regression analysis between age, Df, and muscle mass loss.

		B	SE	Wald	*P*	OR (95%CI)
Model 1	Age	0.139	0.068	4.214	0.040	1.149 (1.006–1.313)
Model 2	Df	−0.541	0.219	6.126	0.013	0.582 (0.379–0.894)
Model 3	Df	−0.525	0.229	5.281	0.022	0.591 (0.378–0.926)

Df, ultrasonic diaphragm excursion during forced deep breathing.

## Data Availability

The datasets used and/or analyzed during the current study are available from the corresponding author upon request.

## Abbreviations

Dq: Ultrasonic diaphragm excursion during quiet breathing
Df: Ultrasonic diaphragm excursion during forced deep breathing
ASM: Appendicular skeletal muscle mass
VC: Vital capacity
FVC: Forced vital capacity
FEV1.0: Forced expiratory volume in one second
MMEF: Maximum midexpiratory flow
PEF: Peak expiratory flow
Pimax: Maximal inspiratory pressure
Pemax: Maximal expiratory pressure
CPET: Cardiopulmonary exercise testing
WRmax: Maximal work rate
VO_2_max: Maximal oxygen consumption
VO_2_max@Kg: Maximal oxygen consumption per kilogram
HRmax: Maximal heart rate
V_E_max: Peak minute ventilation during exercise
V_T_max: Peak tidal volume during exercise
ROC: Receiver-operating characteristic curve
AUC: Area under curve.
